# Management of Epileptic Seizures in Disorders of Consciousness: An International Survey

**DOI:** 10.3389/fneur.2021.799579

**Published:** 2022-01-11

**Authors:** Marie-Michèle Briand, Nicolas Lejeune, Nathan Zasler, Rita Formisano, Olivier Bodart, Anna Estraneo, Wendy L. Magee, Aurore Thibaut

**Affiliations:** ^1^Coma Science Group, Groupe Interdisciplinaire de Génoprotéomique Appliquée (GIGA) Consciousness, University of Liège, Liège, Belgium; ^2^Centre du Cerveau, University Hospital of Liège, Liège, Belgium; ^3^Physical Medicine and Rehabilitation Department, Institut de Réadaptation en Déficience Physique de Québec, Quebec, QC, Canada; ^4^Research Center of the Sacré-Coeur Hospital of Montreal, Montreal, QC, Canada; ^5^DoC Care Unit, Centre Hospitalier Neurologique William Lennox, Ottignies-Louvain-la-Neuve, Belgium; ^6^Institute of NeuroScience, UCLouvain, Brussels, Belgium; ^7^Concussion Care Centre of Virginia, Ltd., Richmond, VA, United States; ^8^Tree of Life Services Inc., Richmond, VA, United States; ^9^Department of Physical Medicine and Rehabilitation, Virginia Commonwealth University, Richmond, VA, United States; ^10^IRCCS, Santa Lucia Foundation, Rome, Italy; ^11^Epileptology Unit, Neurology Department, University Hospital of Liege, Liège, Belgium; ^12^Istituto di Ricovero e Cura a Carattere Scientifico (IRCCS) Fondazione Don Carlo Gnocchi, Florence, Italy; ^13^Neurology Unit, Santa Maria della Pietà General Hospital, Nola, Italy; ^14^Boyer College of Music and Dance, Temple University, Philadelphia, PA, United States

**Keywords:** acquired brain injury (ABI), disorders of consciousness (DoC), epileptic seizure, prophylaxis, treatment, diagnosis, amantadine

## Abstract

Epileptic seizures/post-traumatic epilepsy (ES/PTE) are frequent in persons with brain injuries, particularly for patients with more severe injuries including ones that result in disorders of consciousness (DoC). Surprisingly, there are currently no best practice guidelines for assessment or management of ES in persons with DoC. This study aimed to identify clinician attitudes toward epilepsy prophylaxis, diagnosis and treatment in patients with DoC as well as current practice in regards to the use of amantadine in these individuals. A cross-sectional online survey was sent to members of the International Brain Injury Association (IBIA). Fifty physician responses were included in the final analysis. Withdrawal of antiepileptic drug/anti-seizure medications (AED/ASM) therapy was guided by the absence of evidence of clinical seizure whether or not the AED/ASM was given prophylactically or for actual seizure/epilepsy treatment. Standard EEG was the most frequent diagnostic method utilized. The majority of respondents ordered an EEG if there were concerns regarding lack of neurological progress. AED/ASM prescription was reported to be triggered by the first clinically evident seizure with levetiracetam being the AED/ASM of choice. Amantadine was frequently prescribed although less so in patients with epilepsy and/or EEG based epileptic abnormalities. A minority of respondents reported an association between amantadine and seizure. Longitudinal studies on epilepsy management, epilepsy impact on neurologic prognosis, as well as potential drug effects on seizure risk in persons with DoC appear warranted with the goal of pushing guideline development forward and improving clinical assessment and management of seizures in this unique, albeit challenging, population.

## Introduction

Seizure is a transient clinical event that is characterized by abnormal excessive or synchronous neuronal activity in the brain (1). Epilepsy can be defined as the history of at least one seizure and the presence of an enduring brain condition that increases the likelihood of future seizures (1, 2). Epilepsy can occur secondary to brain lesions (e.g., traumatic brain injury, cerebrovascular disease, central nervous system infections) as classified by the International League Against Epilepsy (2).

For acquired brain injury (ABI), it has been established that there are two types of seizures; acute symptomatic seizures (3) [previously called early post-traumatic seizures (4)] that occur within the first week after the ABI, and unprovoked remote symptomatic seizures (5) (previously called late post-traumatic seizures) that happen after the first week post-injury. At least two unprovoked remote symptomatic seizures that occur more than 24 h apart or after a single event that occurs in a person who is considered to have a high risk of recurrence (>60% risk in a 10-year period) define post-traumatic epilepsy (PTE) after ABI (6). Among ABI etiologies, traumatic brain injury (TBI) is the only one extensively studied in the scientific literature. According to several medical association guidelines such as the American Association of Neurology, the American Academy of Physical Medicine and Rehabilitation, and the Canadian Evidence-based Review of moderate and severe Acquired Brain Injury, acute symptomatic seizures should be prophylaxed with a 1-week course of antiepileptic drug/anti-seizure medication (AED/ASM) treatment such as phenytoin or carbamazepine (7, 8). Research has shown that there are no benefits to continuing prophylaxis after the first week post-injury (9) as it does not prevent the later development of PTE (10–13). On the other hand, several factors influence the risk of developing unprovoked remote symptomatic seizures, such as the occurrence of acute symptomatic seizures or the severity of the brain injury, among other factors (9).

Patients with severe acquired brain injury (ABI) resulting in a disorder of consciousness (DoC) are at high risk of developing seizures due to several different theorized pathoetiologies; however, the prevalence rate is not well established. In a recent survey on diagnostic and prognostic issues in patients with DoC, epilepsy was considered as potentially influencing recovery by 67.7% of the respondents (14). Bagnato et al. reported remote seizures in 32% of patients in unresponsive wakefulness syndrome/vegetative state (UWS/VS—presence of arousal without signs of awareness) and 11% of patients in minimally conscious state (MCS—presence of arousal and fluctuant but reproducible signs of awareness) in the first 3 months post-ABI (15). Pascarella et al. reported 26% (35/130) of patients had remote epileptic seizures and 47% (61/130) had epileptiform activity in a cohort of patients with DoC followed for 30 months after brain injury (16). In this prospective study, no difference between patients in UWS/VS and MCS was shown in terms of ES incidence, nor any association with mortality, whereas ES (whether clinical or subclinical) have clearly been shown to potentially cloud as well as hamper long-term recovery of consciousness (16).

While there are clear diagnostic criteria and treatment guidelines for epilepsy, there is minimal information available specifically for patients with DoC. This lack of information could be explained by the fact that this medical condition is relatively rare in comparison to epilepsy from other etiologies (e.g., genetic or metabolic) (17, 18). Currently, there are no guidelines or recommendations available to help clinicians to diagnose and/or treat epilepsy in this population.

Finally, amantadine, a medication recommended to improve arousal/awareness and neuromotor function in this population (19), has been suggested to lower seizure threshold (20). Yet, there are no studies about this potential association in patients with DoC. Moreover, reports of this kind could lead to omission of this treatment option, potentially compromising a best evidence treatment to improve consciousness.

On the basis of the lack of information and studies about the management of epileptic seizures in patients with DoC, the International Brain Injury Association's DoC Special Interest Group (IBIA'S DOC SIG) conducted an international survey in an effort to identify: (i) the attitudes of clinicians dealing with patients with DoC and ES/PTE, (ii) practice trends in prophylaxis, diagnosis, and treatment, as well as (iii) amantadine use and perceived risks of same relative to seizures.

## Materials and Methods

A questionnaire was developed in 2017 by experts in DoC and/or ES (NL, AT, NZ, RF, AE, OB) targeting what were regarded as the most relevant open questions according to both an extensive review of the evidence-based literature and clinical practice experience.

The survey consisted of 32 questions, including 10 demographic questions and 22 questions related to ES/PTE and DoC. The latter consisted of four questions on ES/PTE prophylaxis, two questions on ES/PTE diagnosis, eight questions on ES/PTE treatment habits and eight on amantadine use in patients with DoC (see Supplementary Material for the complete survey).

The survey was launched using Survey Monkey (SurveyMonkey Inc, San Mateo, California, USA) on March 28th 2018 and disseminated *via* e-mail to all members of the IBIA. Of the 33,295 members of the IBIA contacted, 156 were members of the DOC-SIG and of these, a minority were physicians. We targeted medical doctors because most questions were related to medical management including drug prescription practices. A reminder e-mail was sent on April 17th, 2020 and the survey closed 2 weeks later. Data were exported to Excel (Microsoft, WA, United States) and checked to exclude any duplicates.

## Results

### Demographics

**Figure 1:**
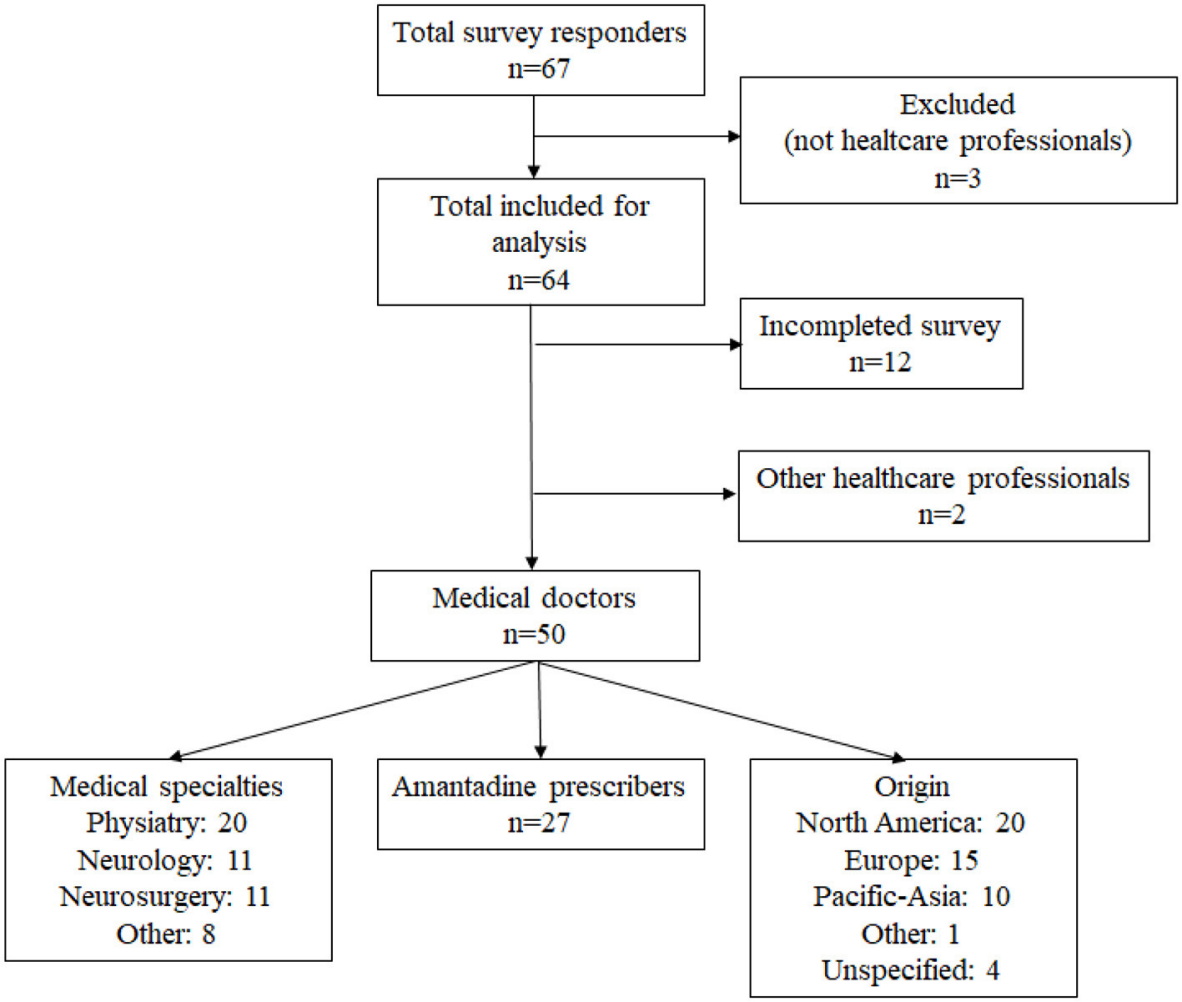
Flowchart of the survey respondents.

Sixty-seven respondents answered the online survey. Three were excluded from the analyses because they were not healthcare professionals (e.g., mother of an epileptic child). After analyses, we excluded respondents who completed less than half of the survey (excluding the demographic questions). The remaining respondents were medical doctors and were included in subsequent analyses (see Figure 1). However, as implied above not all physicians answered all questions, and results are therefore reported on the basis of total responses for each question.

The analyzed sample included 50 medical doctors who hail from almost all continents (see Figure 1) and the majority reported working in post-acute care (23/50–46%) or in acute care facilities (16/50–32%). The other respondents reported working in research units (8/50–16%), private companies (2/50–4%) or in chronic care facilities (1/50–2.0%). They predominantly worked in public practice (31/49–63.2%), had a mix of experience in clinical and research work (30/50–60%), worked with adults (27/50–54%) or all ages (18/50–36%) and had been working for more than 15 years with patients with epilepsy (23/50–46%). Most of the respondents were clinicians (33/50–66%) as opposed to clinician-researchers (17/50–34%). Respondents also had different professional specializations: physiatry (40.0%), neurology (22.0%), neurosurgery (22.0%) and other (16%) and worked in diverse settings and/or with patient populations including intensive care, pediatrics, geriatrics, and psychiatrics (Figure 1). The data examining distinctions between groups relative to years of experience with DOC and/or epilepsy did not yield any clear differences. Additional results are available in the Supplementary Material 2.

### Prophylaxis of ES/PTE

Questions 11–14 explored the participants' habits toward ES prophylaxis. First, the majority of respondents reported using national guidelines (20/47–41.7%) [especially in North America (13/20–65.0%)] or international guidelines (9/47–18.8%). However, up to 16.7% (8/47) responded that they did not use any guideline and 6.3% (3/47) or answered “I don't know” as to which guidelines were used in their medical setting.

Second, several conditions were regarded as a possible indication for prophylactic antiepileptic therapy: severe TBI (27/49–55.1%), intracerebral hemorrhage (19/49–38.8%) and subarachnoid hemorrhage (16/49–32.7). Thirteen out of 49 (26.5%) respondents reported never using prophylactic AED/ASM treatment, regardless of the patient's underlying condition. Third, the preferred AED/AMS was reported to be Levetiracetam (39/48–81.3%) [especially in North America (19/20–95%)], phenytoin (22/48–45.8%) [especially by Pacific-Asian respondents (9/9–100%)], valproic acid (17/48–35.4%), and carbamazepine (7/48–14.6%). Finally, AED/ASM prophylaxis was withdrawn in the absence of evidence of clinical seizure activity (27/47–57.4%), if a sufficient delay since onset was evident (21/47–44.7%), and in the absence of epileptic abnormalities on standard EEG (16/47–34.0%) or on a 24-h EEG (6/47–12.8%). The timeframe of “sufficient delay” was highly variable, as demonstrated by the range of answers, ranging from 1 week (6/21–28.6%), 7–14 days (1/21–4.8%), 3–6 months (4/21–19.0%), to 2 years (1/21–4.8%). Many did not specify the delay (8/21–43%). Multiple answers were permitted for questions 11, 13 and 14.

### Diagnosis of ES/PTE

Questions 17 and 18 inquired about the preferred method and the timing of ES/PTE assessment in the absence of clinical seizure in the subacute or chronic phase. The preferred method was standard EEG (26/46–56.5%), followed by 24-h EEG (5/46–10.9%), none (5/46–10.9%), sleep deprived EEG (4/46–8.7%), monitoring EEG (4/46–8.7%) and video EEG (1/46–2.2%). These tests were ordered when there was lack of neurological progress (25/46–54.3%), when the patient's level of consciousness worsened [according to CRS-R (12/46–26.1%) or by any other measures (17/46–37.0%)], once a year (3/46–6.5%), once every 2 months (3/46–6.5%), once a month (1/46–2.2%), less than once a month (1/46–2.2%) and never (4/46–8.7%). Multiple answers were permitted for the latter question.

### Treatment of ES/PTE

#### Generality

Questions 15 and 16 explored the criteria used to withdraw AED/ASM treatment and to assess its efficacy. Multiple answers were allowed. Treatment efficacy was estimated by the absence of clinical seizure (40/46–87.0%) followed by the reduction of clinical seizure activity (21/46–45.7%) and AED/ASM blood levels (17/46–37.0%). None of the neurologists used the latter criteria (0/10–0%), while half of the neurosurgeons (4/8–50.0%), more than half of the physiatrists (11/20–55.0%) and a third of the other specialty background (2/6–33.3%) used AED/ASM blood levels. Treatment was withdrawn mainly based on the first seizure occurring within 7 days post-onset of the brain injury (21/45–46.7%) and the absence of epileptic abnormalities on standard EEG (17/45–37.8%). The other less frequent answers included: last seizure occurred more than 6 months ago (12/45–26.7%), more than 1 year ago (11/45–24.4%), more than 2 years ago (8/45–17.8%) and the absence of epileptic abnormalities on 24-h EEG (7/48–14.6%). Only a few participants specified other criteria such as age, etiology and time post-brain injury. No one answered that the treatment should be withdrawn if the first seizure occurred in the first month post-onset. Questions 31 and 32 explored the association between treatment and seizure threshold. The risk of the treatment lowering the patient's seizure threshold was assessed by the majority of respondents (30/42–71.4%) before introducing a new treatment especially if the patient was deemed epileptic (8/42–19.0%, total of 90.4%). Respondents were also aware of the risk of lowering seizure threshold with some antibiotics (13/29–44.8%), antipsychotics (13/29–44.8%) and antidepressants (10/29–34.5%).

#### Disorders of Consciousness

Questions 19 to 22 explored the criteria used to start AED/ASM treatment in patients with a DoC, asking about (1) the timing to start AED/ASM treatment in the post-acute phase (after 7 days post-injury), (2) the influence of the level of consciousness, (3) the preferred AED/ASM(s) and (4) the most important factor in AED/AMS choice. The main reason to start AED/ASM therapy was the occurrence of a first clinically evident seizure (36/46–78.3%). The patient's level of consciousness did not influence the majority of the respondents (32/44–72.7%), while 9.0% (4/44) answered “I don't know” and 18.2% (8/44) said they were influenced by the level of consciousness (of which four were neurosurgeons and the other four were from various specialties). Moreover, EEG related criteria, such as the presence of spike/sharp waves, were used assuming they were of high frequency (16/46–34.8%), or regardless of their frequency/distribution (11/46–23.9%). Additionally, the presence of periodic patterns was often selected as a determining factor in AED/ASM initiation (only two out of nine participants that selected this answer mentioned the type of periodic pattern, and these two participants mentioned any periodic epileptiform discharge patterns, lateralized or not). Thirdly, levetiracetam (33/43–76.7%) was the AED/ASM of choice for the treatment of epilepsy in patients with DoC. Valproic acid (20/43–46.5%), carbamazepine (9/43–20.9%), lacosamide (9/43–20.9%) and lamotrigine (9/43–20.9%) were the next most popular options. Five out of 43 respondents (11.6%) reported phenytoin as their preferred AED/ASM. Lastly, the most important criterion guiding the choice of AED/ASM was a low risk of cognitive side-effects (20/42–47.6%). This answer was the most frequent for North American clinicians (11/20–55.0%) and internationally for physiatrists (14/19–73.7%). Other frequent answers were the possibility to quickly titrate up the dose (6/42–14.3%), the general prevalence of adverse side effects (6/42–14.3%) and a large therapeutic drug range (5/42–11.9%).

### Amantadine

Questions 23–30 explored the attitudes toward amantadine treatment and beliefs regarding a possible association with lowering seizure threshold. More than half of the participants (31/43–72.1%) replied that they prescribed amantadine (from always to sometimes) to patients with a DoC. North Americans in the survey used it the most (16/17–94.1%) whereas European usage was less (8/12–66.7%) and Pacific-Asians (4/9–44.4%) even less. All physiatrists (18/18–100.0%) answered in favor of the use of amantadine. The majority of the respondents were not familiar with any literature referring to a causal link between amantadine and seizure (25/43–58.1%). Detailed results according to medical specialty are presented in Table 1. The majority reported that they sometimes (23/43–53.5%) or never (18/43–41.9%) experienced an association between amantadine and seizures in this patient group. Only 4.7% (2/43) endorsed frequently experienced seizures in association with amantadine use in patients with a DoC. Nonetheless, the respondents reported to only sometimes or never using amantadine in ES/PTE patients with a DoC (17/43–39.5% each) or in patients with a DoC with epileptic abnormalities on EEG (15/43–34.9% and 20/43–46.5%, respectively).

**Table 1:** Proportion of respondents to amantadine questions based on medical specialties and their answers.

**Amantadine questions**	**Neurology**	**Neurosurgery**	**Physiatry**	**Others**	**Total**
Proportion of respondents to amantadine questions on total of respondents for each specialty (%)	10/11 (90.9)	9/11 (81.8)	18/20 (90.0)	6/8 (75.0)	43/50 (86.0)
**Amantadine answers**	***n*** **(%)**	***n*** **(%)**	***n*** **(%)**	***n*** **(%)**	***n*** **(%)**
User of amantadine with DoC	6 (60.0)	4 (44.4)	18 (100)	3 (50.0)	31 (72.1)
Know the association with seizure	4 (40.0)	1 (11.1)	9 (50.0)	4 (66.7)	18 (41.9)
Have experienced the association between amantadine and seizure	8 (80.0)	2 (22.2)	13 (72.2)	3 (50.0)	26 (60.5)
User of amantadine in with ES/PTE	5 (50.0)	2 (22.2)	16 (88.9	4 (66.7)	27 (62.8)
User of amantadine in the presence of EA on EEG	4 (40.0)	3 (33.3)	14 (77.8)	3 (50.0)	24 (55.8)

*DoC, disorders of consciousness; EA, epileptic abnormalities; EEG, electroencephalogram; ES, epileptic seizure; PTE, post-traumatic epilepsy*.

Amantadine users were asked about their criteria to determine if it was safe to start amantadine in patients with DoC and ES/PTE. Many answered that they would start amantadine regardless of the time of the last seizure, epileptic abnormalities on EEG, or blood level AED/ASM (12/31–38.7%), while others preferred to first prescribe amantadine when the standard EEG did not demonstrate epileptic abnormalities (10/31–32.3%). Once the decision to prescribe amantadine to an epileptic patient with a DoC is made, most users described their strategy as being to start amantadine at a lower dosage than usual and proceed at a slower incremental titration rate (9/27–33.3%), followed by same dosage with slower incremental titration rate (7/27–25.9%) or the same incremental titration rate as usual (6/27–22.2%). Lastly, in the situation where a seizure occurred while on amantadine, the main strategy adopted by users was to quickly taper the amantadine until full withdrawal (11/29–37.9%), followed by withdrawal without tapering (6/29–20.7%) and tapering with maintenance in the intermediate dosage range (5/29–17.2%).

## Discussion

### Demographics

This study aimed to explore medical doctors' attitudes toward ES/PTE, as well as amantadine use in persons with a DoC following severe acquired brain injury. Given the nature of the survey, physicians were clearly the only appropriate clinical practitioner group to include in this international study. This respondent cohort was limited in number, reflecting the small number of physicians involved in patients with DoC care who were members of the DoC-SIG of the IBIA. In addition, although there was specialty and geographic diversity among respondents, physiatrists (20/50–40%) and United States and Europe (39/50–78%) respectively, were the most represented subgroups. Despite the perception of practice differences between physiatrists and neurologists, they provided parallel responses to the majority of the survey questions. Discrepancies are discussed in the different subsections of the discussion.

### Prophylaxis of Seizures

There are no clear recommendations for post-ABI seizure prophylaxis for many etiologies of acquired brain injury such as ischemic stroke, intracerebral and subarachnoid hemorrhages (21). However, prophylaxis treatment should be applied when the patient is at high risk for acute symptomatic seizure, particularly in the 7-day post-injury period, as for TBI patients (3). Nonetheless, TBI is the only etiology for which there is solid evidence based research examining AED/ASM treatment for seizure prophylaxis for acute symptomatic seizures (7), although the guidelines and recommendations generated from this evidence were not always followed with regard to decisions on initiation of treatment as well as the timing of initiation and termination of treatment (22). In these circumstances, it is not surprising that approximately a quarter of respondents prescribed AED/ASMs contrary to available guidelines or ignored said guidelines.

Phenytoin has been the first-choice prophylactic AED/ASM in patients with TBI (12) but the risk of serious adverse effects (e.g., arrhythmias, severe skin hypersensitivity reaction), drug-drug interactions (23, 24) and the need for serum drug monitoring (25, 26) led to a change toward a more recently developed AED/ASM (i.e. levetiracetam) (27). However, recent reviews suggested that the two AED/ASMs are equivalent in terms of safety and efficacy (24, 28, 29); which seemed to be reflected by the respondents' answers for the use of these two AED/AMSs. Additionally, even though valproic acid has been associated with a possible higher mortality rate (11), it has been reported as frequently used (35.4%) by the respondents. This finding could likely be ascribed to the current use of valproic acid for treating concomitant behavioral disorders, as in patients emerged from DoC and in a confusion state (30, 31).

### Diagnosis of Epilepsy

The European Society of Intensive Medicine recommended the use of continuous EEG monitoring in the acute period, especially in patients in coma with (1) unexplained and persistent altered consciousness to detect non-convulsive seizure or non-convulsive status epilepticus, (2) subarachnoid hemorrhage to detect ischemia when neurological examination is unreliable, and (3) after cardiac arrest to form a precise prognosis (32). Video monitoring EEG has been considered as the gold standard method to diagnose PTE (33). However, these two methods have been recognized as expensive and not easily available (34). Therefore, standard EEG seems to be the method of choice to diagnose epilepsy, according to the guidelines developed by the Royal College of Physicians (RCP) in the UK (35). In patients with DoC, repeated standard EEG recording has been suggested for identifying patients at risk to develop seizures (16), as well as for disentangling UWS/VS from MCS (36). Standard EEG was probably the most used method reported in our survey due to its wide availability and the importance of ruling-out an underlying (non-clinical) seizure occurrence that might hamper the emergence from the DoC (35). Its use was based, for the majority, on the clinical assessment (lack of neurological progress, decrease in Coma Recovery Scale-Revised (CRS-R) score or in level of consciousness) and was not systematic or based on specific time intervals.

### Treatment of Seizures and Epilepsy

Success of AED/ASM therapy has many definitions such as the absence of seizure or seizure frequency reduction by at least 50%. Recently, the Task Force of the International League Against Epilepsy Commission on Therapeutic Strategies proposed that the ability to triple the longest inter-seizure interval could be a good indicator of treatment efficacy (37). So, when questions regarding treatment efficacy criteria were asked, most participants answered that they based their evaluation on clinical features such as a reduction in seizures. In opposition, AED/ASM blood levels have been shown to be useful in compliance evaluation, in assessing drug interactions as well as determining subtherapeutic vs. supratherapeutic drug levels, and in the latter case avoiding toxicity (38). AED/ASM blood levels should not be used to determine the efficacy of the treatment in and of themselves (39). None of the surveyed neurologists selected AED/ASM blood levels as a marker of treatment efficacy.

AED/ASM withdrawal is mostly based on clinical judgement as no guideline has yet addressed this question. For survey participants, seizure in the first 7 days post-trauma seemed to be a good criterion to withdraw AED/ASM as it was the most frequent answer. However, it has been demonstrated that seizure(s) in the first week increase(s) the likelihood to develop epilepsy in the first 2 years following TBI (40) and adding recommendations for the timing of seizures as a second criterion might be essential. No consensus was present for timing across participants as answers varied from 6 to 12 to 24 months almost equally. The second most frequent answer was the absence of epileptic abnormalities on standard EEG, which might suggest lower risk of ES occurrence during AED/ASM withdrawal.

Based on the available literature, it has been shown that patients with DoC are at risk of both seizures and epilepsy (15) and that the frequency seems higher for patients with UWS/VS (32.0%) compared to MCS (10.8%). One study evaluated the impact of AED/ASM treatment on the level of consciousness and outcomes in patients with DoC and found no effect on consciousness recovery 3 months after admission to a post-acute facility and no influence on the number of prescribed AED/ASMs nor the diagnosis (UWS/VS vs. MCS) (41). The answers received in this survey seem to reflect the literature as most of the respondents did not base their decision to treat on the level of consciousness. They mostly started AED/ASM after a first clinical seizure or based on EEG criteria such as the presence of epileptiform discharges. The latter is important as in a study on patients with non-traumatic spontaneous intracerebral hemorrhage, 19% had clinical seizure and an additional 13% had seizure only detected on EEG (42), which underlines the importance of EEG to exclude non-convulsive seizures including status (43). Moreover, the identification of electrographic seizures could help determine at least one of the factors contributing to the DoC (44) and might also influence the decision about AED/ASM use, even if benefit to treat has not been established yet (45). In addition, electrographic seizures have been associated with a poorer neurological outcome in patients with a DoC (46). The more frequently seen patterns in a study examining this in non-traumatic brain injury were periodic epileptiform discharges, periodic lateralized epileptiform discharges and stimulus-induced rhythmic, periodic, or ictal discharges (42). Unfortunately, the role of specific periodic patterns has not yet been determined (46).

Finally, criteria to choose AED/ASMs varied among respondents; physiatrists largely considered the low cognitive impact of the AED/ASM as the most important criteria which differed from other specialties (see Supplementary Material 2). This might be explained by the more frequent involvement of physiatrists in the subacute and chronic care of patients with DoC, periods when patients are generally more medically stable (compared to the acute period) and the focus is on functional rehabilitation. The AED/ASM choice is not benign as it could improve level of consciousness by helping treating ES/PTE or, on the other hand, decrease the level of consciousness secondary to sedative effects (43).

### Amantadine

Since 2012, when a placebo-controlled trial demonstrated the positive effects of a 4-week period of amantadine treatment in patients with severe TBI (47), amantadine has been recommended in different guidelines for use in this population (7, 35). The respondents in this survey seem to be informed by this literature, as most would prescribe amantadine to such patients.

Isolated case reports (48, 49) previously associated amantadine intake with cortical myoclonus and/or ES (50). More recently, amantadine treatment was halted due to continuous epileptic facial myoclonus in one non-traumatic patient in MCS, despite clear behavioral improvement in level of consciousness (20). Nonetheless, in the Giacino et al. placebo-controlled trial (47), there was no difference between the amantadine and the placebo groups for side-effects, including seizure, which could explain why many medical doctors who prescribe amantadine have no knowledge of the possible association with ES or myoclonus. On the other hand, there was also a majority of respondents who answered that they had sometimes experienced that association, which raised concerns regarding the lack of literature on the subject.

### Limits of the Study

Online survey design is an exploratory way to gather information on a topic. In our study, the goal was to reach the maximum number of international medical doctors involved with caring for patients with a DoC. However, we realized that the extent of IBIA community's outreach is limited in South America, Pacific-Asia and Africa. Consequently, we do not have a representative sample for all regions of the world. Plus, all North American respondents were from the USA except one. Physiatrist members are overrepresented compared to other specialties. Questionnaires may also lead to selection bias because only the more motivated/engaged individuals will answer it and answer all questions to the end (51). Finally, the number of respondents is relatively small and limits the generalizability of our findings. In the future, larger surveys should be conducted in collaboration with other associations to reach physiatrists and neurologists outside USA and Europe.

## Conclusion

Our survey demonstrated important discrepancies on how ES/PTE are prevented, diagnosed and treated in patients with a DoC. These results reinforce the lack of literature on this specific topic, poor guideline adherence where applicable and the need for further research on this important area of medical management in persons with a DoC. The same conclusion seems to be true for the use of amantadine with this group of patients. Association between amantadine and ES is still anecdotal and the identification of common characteristics between patients who are at risk to develop seizure with amantadine clearly warrants further study. The population of patients with a DoC is small and those with ES/PTE even smaller. Nonetheless, these patients should benefit from receiving the best quality of care as it could influence their level of awareness and long-term outcomes. It is also likely that clinical decisions related to ES/PTE, AED/ASM therapy and amantadine use could have a long-term impact for both under- and over-treated patients and this is one of the main reasons why further studies are crucial.

## Study Implications Warranting Further Prospective Research

- Prophylaxis: A 7-day period seems to be indicated in patients with severe TBI and in the majority of ABI from other etiologies. Levetiracetam seems to be the AED/ASM of choice.
- Diagnosis: When a lack of neurological progress is observed, a repeated standard EEG is recommended although the gold standard to diagnose epilepsy or epileptic abnormalities is a more prolonged period of testing, 24 h EEG with or without video.
- Treatment: Levetiracetam seems to be the most preferred AED/ASM because of its mild sedating effects, quick titration rate, minimal drug-drug interaction and no monitoring needed. In the case of epilepsy, the period without seizure necessary to start AED/ASM withdrawal remains unknown.
- Amantadine: In the absence of further evidence, amantadine should be used carefully in patients with a DoC deemed to be at significant risk for ES/PTE or with EEG epileptic abnormalities. Low dosage initiation with slow titration rate is recommended if the medication is to be used in such patients.

## Data Availability Statement

The raw data supporting the conclusions of this article will be made available by the authors, without undue reservation.

## Author Contributions

NL, AT, NZ, RF, OB, AE, and WM: conceptualization. NL and AT: methodology, software, and validation. M-MB: formal analysis. M-MB: writing—original draft preparation under AT supervision. NL, NZ, RF, OB, AE, WM, and AT: review and editing. All authors have read and agreed to the published version of the manuscript.

## Funding

M-MB would like to thank the Université Laval (Québec, Canada) for their financial support as she received the McLaughlin award, the Canadian Institute of Health Research (CIHR) and the Fonds de Recherche du Québec-Santé (FRQ-S). All authors thank the University and University Hospital of Liege, the Belgian National Funds for Scientific Research (FRS-FNRS), the European Union's Horizon 2020 Framework Programme for Research and Innovation under the Specific Grant Agreement No. 785907 (Human Brain Project SGA2), the Luminous project (EU-H2020-fetopenga686764), the European Space Agency (ESA) and the Belgian Federal Science Policy Office (BELSPO) in the framework of the PRODEX Programme, the Center-TBI project (FP7-HEALTH-602150), the Public Utility Foundation Université Européenne du Travail, Fondazione Europea di Ricerca Biomedica, the Bial Foundation, the Mind Science Foundation and the European Commission, the fund Generet, the King Baudouin Foundation, the Mind-Care foundation, DOCMA project (EU-H2020-MSCA–RISE−778234). AT is Research Associate and NL a Post-Doctoral Researcher at the FNRS.

## Conflict of Interest

The authors declare that the research was conducted in the absence of any commercial or financial relationships that could be construed as a potential conflict of interest.

## Publisher's Note

All claims expressed in this article are solely those of the authors and do not necessarily represent those of their affiliated organizations, or those of the publisher, the editors and the reviewers. Any product that may be evaluated in this article, or claim that may be made by its manufacturer, is not guaranteed or endorsed by the publisher.
