# Supplementary material for: Management of Epileptic Seizures in Disorders of Consciousness: An International Survey
Source: Front Neurol. 2022 Jan 11;12:799579. doi: 10.3389/fneur.2021.799579 (PMC8788407; doi:10.3389/fneur.2021.799579)
Supplement: Supplementary file 1 [file Data_Sheet_1.DOCX]

Supplementary Material

**SURVEY ON EPILEPSY MANAGEMENT IN DISORDERS OF CONSCIOUSNESS (DOC)**

While epilepsy is quite a frequent condition among patients with acquired brain injury, to date, no guidelines nor consensus on epilepsy management exist in the specific case of patients with Disorders of Consciousness (DOC). This international survey aims to explore the specific treatment approaches that are currently being used in different settings.

We ask you to answer each question in relation to your own healthcare institution. Always keep in mind, unless it is explicitly stated, that all the questions relate only to people with DOC (Unresponsive Wakefulness State/Vegetative State, Minimally Conscious State). Feel free to give any comments for all questions.

Thank you for your participation.

1. Professional background (**multiple answers possible**)
   1. Neurologist (or physician extender)
   2. Neurosurgeon (or physician extender)
   3. Physiatrist (or physician extender)
   4. Psychiatrist (or physician extender)
   5. Other (**specify**)
2. Country
3. City/Town
4. Place of Work (please indicate **your** **main work setting**)
   1. Acute care (ICU, neurology ward)
   2. Post-acute care (rehabilitation…)
   3. Chronic care setting (nursing home…)
   4. Research unit (academic, university, research laboratory…)
   5. Other (**specify**)
5. Setting (please indicate **your main work setting**)
   1. Private
   2. Public
6. What is the age range of the patients you handle?
   1. Adult
   2. Pediatrics
   3. Both
7. Experience of working with patients with a DOC?
   1. Clinical only
   2. Research only
   3. Clinical and research
   4. Other (**specify**)
8. Number of years working with DOC
   1. < 2 years
   2. 2-5 years
   3. 6-15 years
   4. > 15 years
9. Experience of working on epilepsy?
   1. Clinical only
   2. Research only
   3. Clinical and research
   4. Other (**specify**)
10. Number of years working on epilepsy?
    1. < 2 years
    2. 2-5 years
    3. 6-15 years
    4. > 15 years
11. In your experience, in which condition(s) is/are prophylactic antiepileptics used in the acute phase (e.g. treatment in absence of clinical evidence of epileptic seizure and/or epileptic abnormalities)? **(multiple answers possible)**

- Severe TBI
- Post-anoxic coma
- Intracerebral hemorrhage
- Subarachnoid hemorrhage
- Metabolic/Infectious etiology
- Others
- Never

1. In your setting, what guideline(s), if any, are used for AED prophylaxis?

- Local guidelines
- National guidelines
- International guidelines
- No guidelines

1. In your setting, which AEDs are MAINLY used for prophylaxis? **(multiple answers possible)**

- Carbamazepine
- Clobazam
- Lacosamide
- Levetiracetam
- Midazolam
- Phenobarbital
- Phenytoin
- Valproic acid
- Other (**please specify**)

1. When do you stop (or begin to withdraw) prophylactic treatment? **(multiple answers possible)**

- Sufficient delay since onset (**if ticked, please specify**)
- Absence of clinical seizure
- Absence of epileptic abnormalities on standard EEG
- Absence of epileptic abnormalities on 24h EEG

1. In the absence of clinical seizure and premorbid history of epilepsy, what criteria do you use to withdraw AED treatment? **(multiple answers possible)**

- First seizure occurred in the first 7 days post-onset

- First seizure occurred in the first month post-onset

- Last seizure occurred more than 6 months ago

- Last seizure occurred more than a year ago

- Last seizure occurred more than two years ago

- Absence of epileptic abnormalities at standard EEG

- Absence of epileptic abnormalities at 24H EEG

- Age (**please specify**)

- Time post-brain injury (**please specify**)

- Etiology **(please specify**)

1. How do you assess the efficacy of the treatment? **(multiple answers possible)**

- AED blood level
- Reduction of epileptic abnormalities to standard EEG
- Absence of epileptic abnormalities to standard EEG
- Reduction of epileptic abnormalities to 24H EEG
- Absence of epileptic abnormalities to 24H EEG
- Clinically (absence of clinical seizures)
- Clinically (reduction of clinical seizures)
- Other (**please specify**)

1. In the subacute and chronic phase of brain injury **what** method do you use to assess for epilepsy in the absence of clinically evident seizure(s)?

- Standard EEG
- Sleep deprived EEG
- 24H EEG
- EEG monitoring
- Other (**please specify)**
- None

1. In the subacute and chronic phase of brain injury, **when** do you assess for epilepsy in the absence of clinically evident seizures in a patient **with a DOC**? **(multiple answers possible)**

- Once a month
- Once every two months
- Once a year
- Less than once a year
- When there is a lack of neurological progress
- When CRS-R scores decreases
- When level of consciousness lowers, measured by any other measures than CRS-R
- Never

1. When do you start AED treatment in patients in DOC during post-acute phase (i.e. after the 7 days post-insult)? **(multiple answers possible)**

- At first clinically evident seizure
- After at least two clinically evident seizures
- In presence of spike/sharp waves regardless of their frequency/or distribution
- In presence of highly frequent spike/sharp waves
- In presence of periodic patterns (please specify : PLEDs/BiPLEDs/GPEDs/IRDA/SIRPIDs)
- Other (**please specify**)

1. Which AED do you prefer to use in patients with prolonged DOC in the post-acute period of recovery for epilepsy management **(multiple answers possible?)**

- Carbamazepine
- Clobazam
- Gabapentin
- Lacosamide
- Lamotrigine
- Levetiracetam
- Midazolam
- Oxcarbazepine
- Phenobarbital
- Phenytoin
- Pregabalin
- Valproic acid
- Other (**please specify**)

1. In your practice, what is the most important factor for choosing an AED for epilepsy management in patients with DOC?

- Large therapeutic range of the drug
- Possibility to quickly titrate up the dose
- General prevalence of adverse side effects
- Low cognitive impact
- Low interactions with other drugs
- Additional expected effect (eg. pain relief, behavioral effect)
- Other: (**please specify**)

1. In general, regarding the questions of the questionnaire, would you handle the treatment differently according to the level of consciousness (coma/VS versus MCS)?

- Yes
  - If your answer is “yes”, how differently would you handle it?
- No
- I don’t know

1. Do you use amantadine for your patients in DOC?

- Yes, always
- Yes, often
- Yes, frequently
- Yes, sometimes
- Never

1. Are you familiar with literature demonstrating a causal link between amantadine and seizures (in general), and in relation to patients with brain injury (or specifically in DOC)?

- Yes
  - If yes, would you please, give us some references?
- No

1. In your experience, how frequently do patients receiving amantadine experience seizures?

- Always
- Often
- Frequently
- Sometimes
- Never

1. Would you or do you use amantadine in **epileptic** patients with a DOC?

- Never
- Sometimes
- Frequently
- Often
- Always, regardless of epilepsy

1. Would you or do you use amantadine in DOC patients with epileptic abnormalities on EEG?

- Never
- Sometimes (**please specify**)
- Always, regardless of epileptic abnormalities

1. If you use amantadine in DOC patients with epilepsy, how do you decide to start it? **(multiple answers possible)**

- Last seizure occurred more than 6 weeks ago
- Last seizure occurred more than 6 months ago
- No epileptic abnormalities on standard EEG
- No epileptic abnormalities on 24h EEG
- With blood level of AED in the therapeutic range
- Regardless of these statements.
- Other (**please specify**)

1. If you decide to give amantadine to a DOC patient with epilepsy, how do you initiate treatment?

- Same dosage as usual, but slower increase
- Same dosage as usual, same incremental titration rate
- Lower dosage than usual and slower incremental titration rate
- Lower dosage than usual but same incremental titration rate
- Increase as regard to the clinical or EEG monitoring
- In increasing the AED dosage

1. If your patient develops seizures while being given amantadine, what do you do? **(multiple answers possible)**

- Slow tapering until withdrawal
- Quick tapering until withdrawal
- Withdrawal without tapering
- Tapering and maintain intermediate dosage
- Maintain same dosage
- Increasing or adding AED
- Other (**please specify**)

1. Before introducing a new treatment, do you ascertain the risk that the treatment will not lower the patient’s seizure threshold?

- Yes, always

- Yes, but only if the patient has epilepsy

- Yes, if it will be in combination with other drugs lowering the seizure threshold

- Sometimes (**please specify**)

- No

1. What are, in your experience, the drugs that most frequently lower seizure threshold in patients with DOC, due either to their frequency of use or of their propensity to induce seizures?

- * Up to five answers, in INN (International Nonproprietary Name)
- …

*This survey is the result of the work of the epilepsy working group of the DOC-SIG treatment group, led by Nicolas Lejeune and Aurore Thibaut. The following members contributed actively to this work: Nathan Zasler, Olivier Bodart, Rita Formisano, Anna Estraneo, Wendy Magee, and Efthymios Angelakis.*
