# Supplementary material for: Management of Epileptic Seizures in Disorders of Consciousness: An International Survey
Source: Front Neurol. 2022 Jan 11;12:799579. doi: 10.3389/fneur.2021.799579 (PMC8788407; doi:10.3389/fneur.2021.799579)
Supplement: Supplementary file 2 [file Data_Sheet_2.DOCX]

Supplementary Materials 2

**1. Responses to question 14, 15, 16, 18. 19 and 21 according to the medical specialties.**

When do you stop (or begin to withdraw) prophylactic treatment? (multiple answers possible)

In the absence of clinical seizure and premorbid history of epilepsy, what criteria do you use to withdraw (multiple answers possible)

How do you assess the efficacy of the treatment? (multiple answers possible) (already discussed)

In the subacute and chronic phase of brain injury, when do you assess for epilepsy in the absence of clinically evident seizures in a patient with a DOC? (multiple answers possible)

When do you start AED treatment in patients with a DOC during post-acute phase (i.e. after the 7 days post- insult)? (multiple answers possible)

What is the most important factor for choosing an AED for epilepsy management in patients with a DOC? (one answer only)

**2. Responses to question 14, 15, 16, 18. 19 and 21 according to year of experience.**

When do you stop (or begin to withdraw) prophylactic treatment? (multiple answers possible)

In the absence of clinical seizure and premorbid history of epilepsy, what criteria do you use to withdraw an AED (multiple answers possible)

How do you assess the efficacy of the treatment? (multiple answers possible) (already discussed)

In the subacute and chronic phase of brain injury, when do you assess for epilepsy in the absence of clinically evident seizures in a patient with a DOC? (multiple answers possible)

When do you start AED treatment in patients with a DOC during post-acute phase (i.e. after the 7 days post- insult)? (multiple answers possible)

When do you start AED treatment in patients with a DOC during post-acute phase (i.e. after the 7 days post- insult)? (multiple answers possible)

What is the most important factor for choosing an AED for epilepsy management in patients with a DOC? (one answer only)
